# Generating a host range-expanded recombinant baculovirus

**DOI:** 10.1038/srep28072

**Published:** 2016-06-20

**Authors:** Chunfeng Wu, Zihao Deng, Zhao Long, Yi Cai, Zhongfu Ying, Hanqi Yin, Meijin Yuan, Rollie J. Clem, Kai Yang, Yi Pang

**Affiliations:** 1 State Key Laboratory of Biocontrol, Sun Yat-sen University, Guangzhou 510275, China; 2 Liuzhou People’s Hospital, Liuzhou 545006, China; 3 Division of Biology, Kansas State University, Manhattan, KS66506, USA

## Abstract

As baculoviruses usually have a narrow insecticidal spectrum, knowing the mechanisms by which they control the host-range is prerequisite for improvement of their applications as pesticides. In this study, from supernatant of culture cells transfected with DNAs of an Autographa californica multiple nucleopolyhedrovirus (AcMNPV) mutant lacking the antiapoptotic gene *p35* (vAc^∆P35^) and a cosmid representing a fragment of Spodoptera exigua nucleopolyhedrovirus (SeMNPV), a viral strain was plaque-purified and named vAcRev. vAcRev had a broader host range than either vAc^∆P35^ or SeMNPV parental virus, being able to infect not only the permissive hosts of its parental viruses but also a nonpermissive host (*Spodoptera litura*). Genome sequencing indicated that vAcRev comprises a mixture of two viruses with different circular dsDNA genomes. One virus contains a genome similar to vAc^∆P35^, while in the other viral genome, a 24.4 kbp-fragment containing 10 essential genesis replaced with a 4 kbp-fragment containing three SeMNPV genes including a truncated *Se-iap3* gene. RNA interference and ectopic expression assays found that *Se-iap3* is responsible for the host range expansion of vAcRev, suggesting that *Se-iap3* inhibits the progression of apoptosis initiated by viral infection and promotes viral propagation in hosts both permissive and non-permissive for AcMNPV and SeMNPV.

Baculoviruses are insect pathogens commonly encountered in nature where they specifically infect insect hosts, mainly lepidopteran insects^1^. As baculoviruses are highly host specific and non-pathogenic to non-target organisms^2^,^3^, they have been used for integrated pest management as microbial pesticides. However, the fact that baculovirus infection is usually limited to a single or a few closely related insect species makes it less attractive economically. To improve the practical effectiveness of baculoviruses, a variety of studies have been conducted regarding the host range determining factors of baculoviruses and how they work.

*Autographa californica multiple nucleopolyhedrovirus* (AcMNPV) is the most widely researched member of the *Baculoviridae* family. Since the budded form of baculoviruses can enter a wide variety of invertebrate and vertebrate cells, host range is not determined at the level of receptor binding. Instead, earlier studies have reported that several AcMNPV genes such as *p143 (helicase)*, *hrf-1*, *hcf-1*, *ie2 and p35* are involved in host range determination^4^,^5^. The blockage of virus infection may vary at different stages in different virus-insect systems, but occur mainly at the steps of transport to the intracellular site of replication, viral gene expression, and generation of viral progeny^6^,^7^,^8^,^9^,^10^,^11^.

In addition, apoptosis of host cells induced by virus infection plays an important role in baculovirus host range. For example, AcMNPV infection induces apoptosis of *Spodoptera litura* or *Spodoptera littoralis* cells^12^,^13^,^14^. *Spodoptera exigua nucleopolyhedrovirus* (SeMNPV) is another member of the *Baculoviridae* family and strains of SeMNPV have a high degree of specificity and infectivity against *S. exigua* both *in vivo* and *in vitro*^15^,^16^,^17^. However, due to apoptosis, SeMNPV replication is restricted in *S. litura* (SpLi-221 and Sl-ZSU-1) cells, *S. littoralis* (CLS-79) cells, and *Lymantria dispar* (Ld652Y) cells^15^,^18^.

Caspases are a family of cysteine proteases that play essential roles in apoptosis^19^. During productive infection, baculoviruses interfere with apoptosis by expressing the apoptotic inhibitors P35/P49 or IAPs^20^. P35 is able to inhibit effector caspases, while P49, a P35 homolog, has similar predicted three-dimensional structure to P35 and inhibits initiator and effector caspases by the same mode of action used by p35 to inhibit effector caspases^21^. The other types of baculovirus anti-apoptotic proteins are IAP proteins, which are unrelated to P35/P49. IAPs contain two main motifs: one to three copies of the BIR (baculoviral IAP repeat) domain at their N-termini and a RING domain near their C-termini^6^,^22^,^23^.

*S. frugiperda* (Sf9) cells and *S. exigua* (Se301) cells infected with AcMNPV mutants that lack the *p35* gene undergo apoptosis, and thus the viral infections are aborted. The *Sl-p49* gene from Spodoptera littoralis nucleopolyhedrovirus (SlNPV) and *iap3* genes from Cydia pomonella granulovirus (CpGV) and Origia pseudotsugata nucleopolyhedrovirus (OpMNPV) are able to substitute for *p35* to rescue AcMNPV replication in Sf9 cells which are permissive to the virus^24^,^25^, demonstrating that anti-apoptosis genes are important factors to affect baculovirus host range. However, although over-expression of the AcMNPV *p35* gene could inhibit apoptosis in AcMNPV infected-*S. littoralis* cells which are non-permissive to the virus, it cannot restore viral replication^26^. Similar results were observed in *S. litura* cells which were infected with a recombinant AcMNPV in which *p35* was substituted by the *p49* gene of SpltNPV which is able to replicate in the cells^27^. These studies indicate that the cellular apoptotic response is not the only limiting factor for host-range in some cases.

Here we obtained a host-expanded viral strain (named vAcRev) by cotransfection of Sf9 cells with an AcMNPV mutant lacking *p35* (vAc^∆P35^) and a DNA fragment of SeMNPV. Besides Hi5 and Se301 cells, which are permissive to vAc^∆P35^ and SeMNPV, respectively, Sf9 cells (semi-permissive to vAc^∆P35^ but nonpermissive to SeMNPV) and SpLi-221 cells (nonpermissive to both parental viruses) are able to support the replication of vAcRev. Furthermore, vAcRev is infectious to *Trichoplusia ni* and *S. exigua* larvae by oral inoculation, and to *S. litura* larvae by hemocoelic, but not oral, inoculation. We further identified that a truncated SeMNPV *iap* gene (*Se-iap3*) is involved in the new traits of vAcRev. Finally, we found that Se-IAP3 inhibits the progression of apoptosis of SpLi-221 cells induced by vAc^∆P35^ infection and supports vAc^∆P35^ proliferation. Considering our previous research in which overexpression of Splt-P49 cannot support vAc^∆P35^ replication in SpLi-221 cells, even though the overexpression blocks virus-induced apoptosis^27^, our finding suggests that IAP3 has an additional unknown function that facilitates viral replication.

## Results

### Obtaining a viral strain with a broader *in vitro* host range

**Figure 1:**
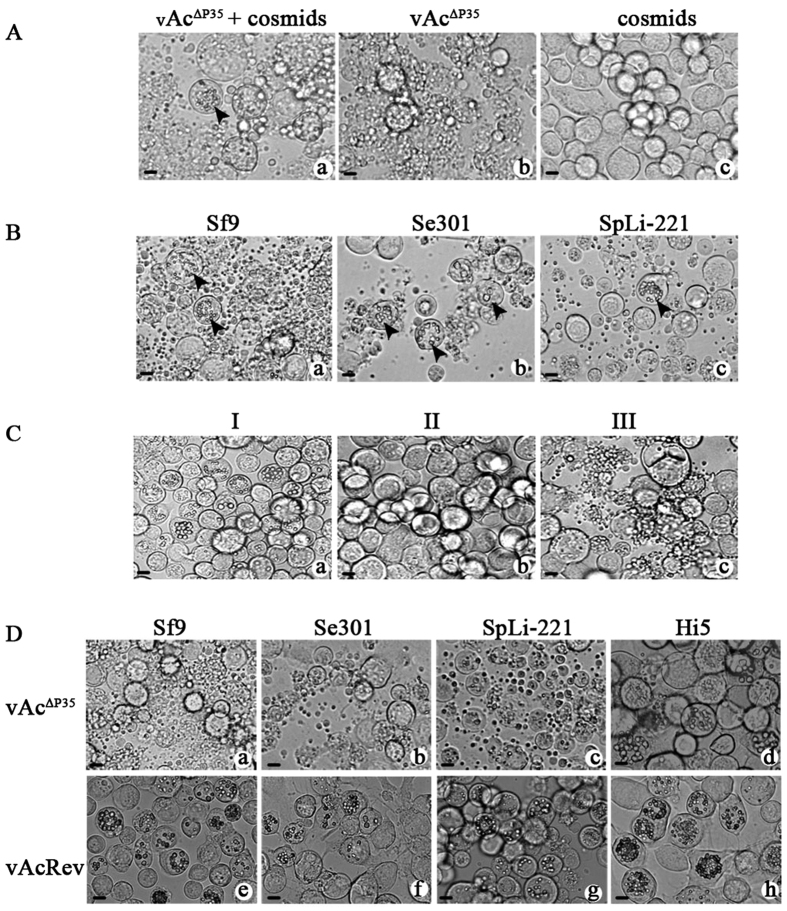
(**A**) Sf9 cells were cotransfected with vAc^∆P35^ and the SeMNPV cosmid library (a). Most transfected cells underwent apoptosis while PIBs were produced in a few cells (arrowhead). Transfection of Sf9 cells with vAc^∆P35^ (b) resulted in cell apoptosis, while no cytopathic effects were observed in the SeMNPV cosmid library-transfected cells (c). Pictures were taken at 5 d p.t. (**B**) Sf9, Se301, and SpLi-221 cells were inoculated with the supernatants from the cotransfected cells. (**C**) Three types morphology of plaques in SpLi-221 cells (see text for description). (**D**) vAc^∆P35^- or vAcRev-infected Sf9, Se301, SpLi-221, and Hi5 cells at 72 h p.i.

To study the potential of the SeMNPV genome sustaining the replication of vAc^∆P35^, vAc^∆P35^ DNA and a SeMNPV cosmid library, which consists of 5 cosmids and represents the entire viral genome^28^, were cotransfected into Sf9 cells. Although most of the transfected cells underwent apoptosis, polyhedral inclusion bodies (PIBs) were observed in a few cells, indicating that productive infection was established in these cells (Fig. 1Aa, arrowhead). By contrast, vAc^∆P35^-transfected Sf9 cells underwent apoptosis severely and no PIBs were formed (Fig. 1Ab); whereas no cytopathic effects were observed in the SeMNPV cosmid library-transfected cells 5 days post transfection (p.t.) (Fig. 1Ac). Surprisingly, when Sf9, SpLi-221, and Se301 cells were inoculated with the supernatants from the cotransfected cells, PIBs were produced in a few cells of all three cell lines (Fig. 1B, arrowheads), although most cells underwent apoptosis. These results indicated that progeny budded virions (BVs) were present in the supernatants. Because, consistent with previous studies, vAc^∆P35^ infection induced apoptosis of Sf9, SpLi-221, and Se301 cells, but not Hi5 cells (Fig. 1D, upper panels), and SeMNPV infection induces apoptosis of SpLi-221 cells, the productive infection of these cells suggested that a viral strain with a broader host range was generated in the cotransfected cells. With 10 undiluted serial passages of the supernatant in Sf9, SpLi-221 and Se301 cells, the percentage of PIB-containing cells increased, and apoptosis was alleviated gradually (data not shown). Apparently, the PIB-positive viral strain became predominant and the proportion of apoptosis-inducing viruses decreased upon serial passage.

Sf9 cells were also cotransfected with vAc^∆P35^ DNA and individual cosmids of the SeMNPV cosmid library. Only cosmid 22 could rescue the replication of vAc^∆P35^ in Sf9 cells, while cells underwent severe apoptosis upon the cotransfection of vAc^∆P35^ DNA with the other individual cosmids (data not shown). The results indicated that one or more genes in cosmid 22 could not only substitute the function of *p35* to block apoptosis, but also allow virus replication in the semi- or non-permissive cell lines for AcMNPV.

Subsequently, plaque purification was carried out to isolate the PIB-positive viral strain. During the purification, three types of plaques were observed: Type I, PIB-positive; Type II, PIB-negative but with cytopathic effect; Type III, cells in the plaque underwent apoptosis (Fig. 1C). Notably, if the inoculum was diluted too much, only Type П and/or Type III plaques were formed. After four rounds of plaque purification focusing on Type I plaque, a viral strain was obtained and designated as vAcRev. vAcRev could stably establish productive infection in Sf9, Se301, SpLi-221 and Hi5 cell lines (Fig. 1D, lower panels). All four cell lines infected with vAcRev showed typical NPV cytopathology, including nuclear hypertrophy, matrix detachment and PIB formation. PIBs became clearly visible at 24 hour post infection (h p.i.) in SpLi-221 cells, 36 h p.i. in Sf9 and Hi5 cells, but only after 48 h p.i. in Se301 cells. The percentage of PIB-containing Sf9, Se301, SpLi-221 and Hi5 cells were 24%, 4%, 29%, and 56% respectively, at 48 h p.i (based on counting about 1000 cells). At 72 h p.i., most of the infected SpLi-221 cells lost adherence, while the other cell lines remained adhered. The percentage of PIB-containing cells was the highest (79%) for Hi5 cells at 72 h p.i., followed by SpLi-221 cells (66%), Se301 cells (48%), and the least for Sf9 cells (49%). Obviously, vAcRev has a wider host range at the cellular level compared with the parent viruses, vAc^∆P35^ and SeMNPV.

### Comparisons of replication between vAcRev and wild type viruses *in vitro* and *in vivo*

**Figure 2:**
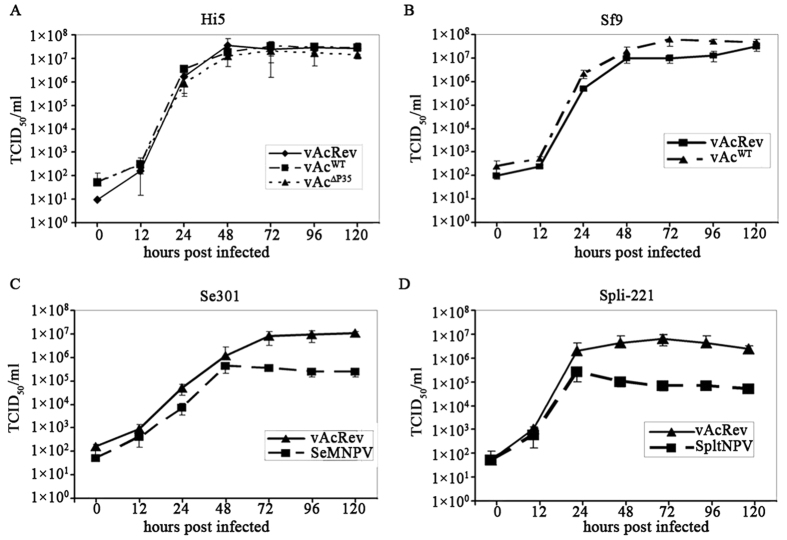
Viral replication kinetics. Cells were infected with the indicated viruses at an MOI of 5 TCID_50_/cell, and the infectious BVs were titered using the corresponding cells. (**A**) vAcRev, vAc^WT^ and vAc^∆P35^ in Hi5 cells. (**B**) vAcRev and vAc^WT^ in Sf9 cells. (**C**) vAcRev and SeMNPV in Se301 cells. (**D**) vAcRev and SpltNPV in SpLi-221 cells. Each datum point was determined from the average of three independent infections and error bars represent the standard errors.

To determine any differences in the replication kinetics between vAcRev and wild type (wt) viruses in corresponding cells permissive to the wt viruses, growth curve assays were performed. There was no significant difference in replication kinetics among vAcRev, vAc^WT^ and vAc^∆P35^ in Hi5 cells (Fig. 2A). The replication kinetics between vAcRev and vAc^WT^ were similar in Sf9 cells (Fig. 2B). In contrast, the yields of infectious BV of SeMNPV and SpltNPV were around two logs lower than that of vAcRev in Se301 (Fig. 2C) and SpLi-221 (Fig. 2D) cells respectively, suggesting that the replication of vAcRev is higher than the two wt viruses in their host cell lines.

Bioassays showed that vAcRev could orally infect *T. ni* larvae; however, different symptoms were observed among the larvae infected with vAcRev, vAc^∆P35^ and AcMNPV. The larvae that died of vAcRev or AcMNPV infection showed typical symptoms of baculovirus infection, but the larvae infected with vAc^∆P35^ did not undergo liquefaction, which is consistent with a previous study^29^,^30^. The LD_50_ values of vAcRev, vAc^∆P35^ and AcMNPV were 82, 141.1 and 111.1 PIBs/larvae respectively, and the values were not significantly different between AcMNPV and vAc^∆P35^ (P = 0.1256) or AcMNPV and vAcRev (P = 0.2914) (Table 1). However, the LD_50_ value for vAcRev was significantly lower than that for vAc^∆P35^ (P = 0.0166).

Bioassays showed that vAcRev could orally infect *S. exigua* larvae, and no significant difference in infectivity was observed between AcMNPV, vAc^∆P35^ and vAcRev, with the LD_50_ values being 4219.4, 6214.2 and 5183.8 PIBs/larvae, respectively (Table 1). However, these viruses were not as infectious in *S. exigua* larvae as SeMNPV, which had an LD_50_ value of 166.0 PIBs/larvae. Larvae that died after infection with AcMNPV or SeMNPV showed typical symptoms of baculovirus infection. In contrast, the dead larvae infected with vAcRev and vAc^∆P35^ did not undergo liquefaction, but exhibited other symptoms of baculovirus infection.

The oral infectivity of vAcRev and vAc^∆P35^ was tested in *S. litura* larvae by infecting with a high concentration of virus (5 × 10^9^ PIBs/ml), but all the larvae pupated, suggesting that vAcRev and vAc^∆P35^ cannot orally infect *S. litura*. Thus, the virulence of the vAcRev and vAc^∆P35^ BV were assayed in *S. litura* larvae by intrahemocoelic injection (Table 1). *S. litura* was susceptible to fatal infection with vAcRev, but the larvae injected with vAc^∆P35^ remained healthy and survived to pupation. The larvae injected with vAcRev did not melt, liquefy or melanize, in contrast to injection with SpltNPV. In addition, the LD_50_ value for vAcRev was approximately 275-fold higher than that of SpltNPV (Table 1).

### Sequencing and analyzing the vAcRev genome

**Figure 3:**
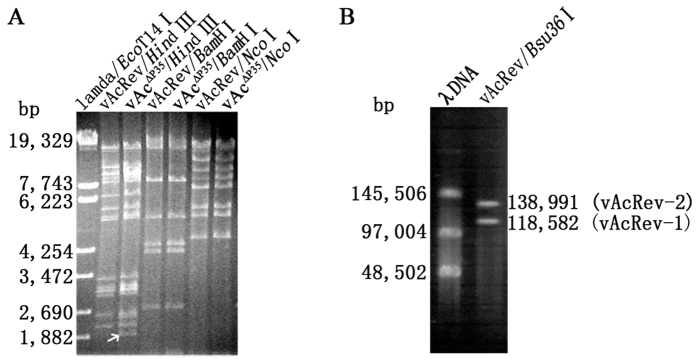
Restriction enzyme analysis of vAcRev genomic DNA. (**A**) Extracted DNA of vAcRev and vAc^∆P35^ were digested with *Hin*dIII, *Bam*HI or *Nco*I, and the digested fragments were separated on 0.7% agarose gel. *Eco*T14I-digested λDNA was used as molecular size standards. (**B**) vAcRev genomic DNA was digested with *Bsu*36I and was analyzed by using pulsed field gel electrophoresis. λDNA was used as a marker.

To investigate any recombination events that may have occurred in the production of vAcRev, genomic DNA from vAcRev was analyzed with restriction endogenous enzymes. Digestion of vAcRev with *Bam*HI or *Nco*I revealed overall restriction enzyme patterns similar to that of vAc^∆P35^, while digestion with *Hin*dIII revealed some deletions and/or insertions (Fig. 3A). These results indicated that vAcRev and vAc^∆P35^ share similar overall genomic structure.

The vAcRev genome was further sequenced by a massive parallel pyrosequencing technology (454 GS-FLX). A total of 127,575 high-quality reads with an average read length of 251 bp were produced. The total number of sequenced bases was 32,058,578 bp, which provided more than 100-fold coverage of the AcMNPV-C6 genome. Assembly performed by the Newbler software of the 454 suite package (454 Life Sciences) resulted in 9 large (defined as >500 bp) contigs. The genome of AcMNPV-C6 was used as a reference to find a proper layout for the contigs. Gaps were filled through sequencing of PCR products by primer walking or specific oligonucleotide primers targeting contig ends between two adjacent contigs. The resulting sequence data were assembled into two circular dsDNA molecules. The results suggest that vAcRev contains two distinct circular dsDNA genomes. One genome was 118,582 bp in length and was designated as vAcRev-1 (GenBank accession no. KU697902), while the other was 138,991 bp and was designated as vAcRev-2 (GenBank accession no. KU697903).

**Figure 4:**
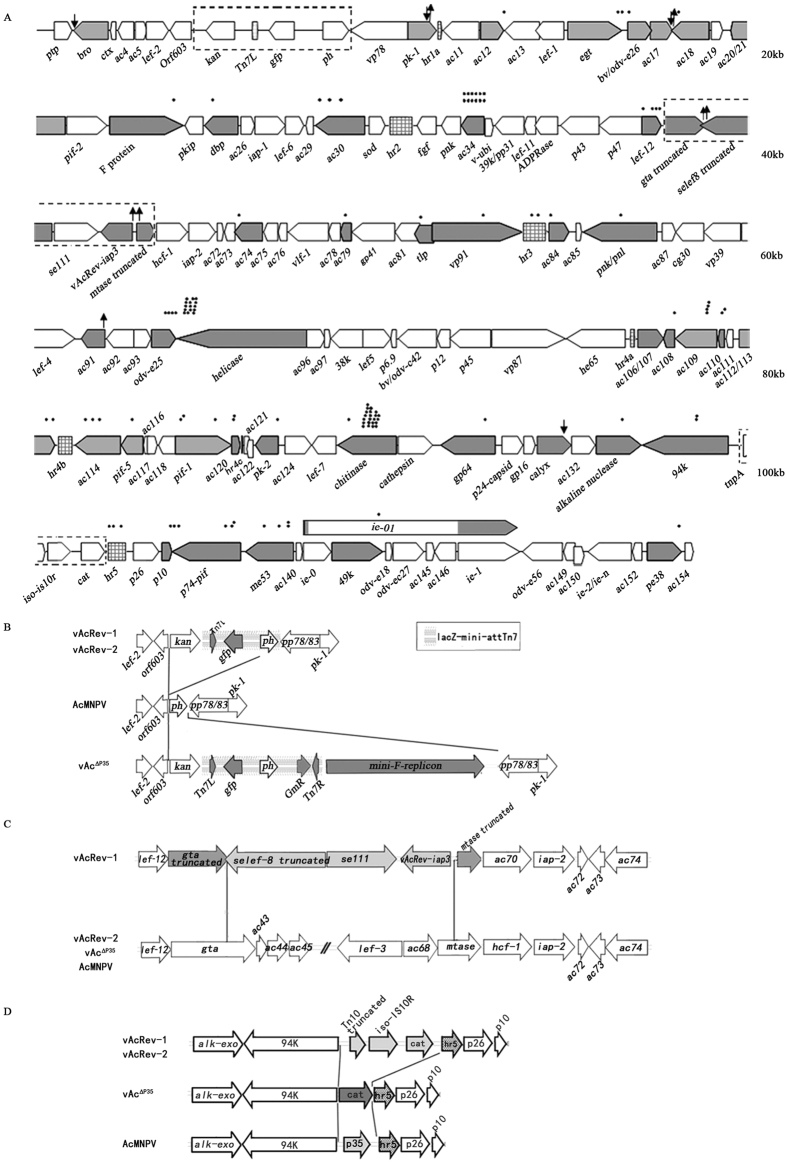
(**A**) Organization of the vAcRev-1 genome. The circular genome is showed in a linear format. The positions of the 131 ORFs identified are indicated by arrows that also represent the direction of transcription. Open arrows indicate that the ORF has 100% amino acid identity to its homologue in AcMNPV. Grey arrows represent that there are deletions, insertions or substitutions compared with its AcMNPV homologue. Dots above the ORFs represent nucleotide substitutions occurred in this regions. ↓ and ↑ represent deletions or insertions, respectively. Highly variable regions are boxed by border lines and the comparisons with the corresponding regions of vAc^∆P35^ and AcMNPV are illustrated in **B~D**. Gene organization in the lef2-pk-1 (**B**), Ac43-68 (**C**), and 94 K-hr5 (**D**) region of vAcRev and the corresponding regions of vAc^∆P35^ and AcMNPV.

By using Vector NTI (Invitrogen) and the AcMNPV-C6 and SeMNPV genomic sequences in NCBI database, ORFs in the vAcRev-1 and vAcRev-2 genomes were predicted. 134 ORFs and 8 hrs in vAcRev-1and 157 ORFs and 9 hrs in vAcRev-2 were identified (Table S1 in the supplemental materials). The organization of the vAcRev-1circular genome is diagrammed in a linear format in Fig. 4A. The variations among vAcRev-1, vAcRev-2, vAc^∆P35^, and AcMNPV are also illustrated in Fig. 4B–D. The major distinction between the vAcRev-1 and vAcRev-2 genomes is a region where a 24.4-kb fragment in vAcRev-2, which contains 26 ORFs (*Ac43~Ac68*), is replaced with a 4.0-kb fragment which is derived from cosmid 22 and contains three ORFs (a truncated *Se-iap3*, *Se111* and a truncated *Se-lef8*) (Fig. 4C). The difference in size between the vAcRev-1 and vAcRev-2 genomes (118,582 versus 138,991 bp) is attributed mainly to this region (4015 versus 24424 bp). The 24.4-kb region contains 10 essential genes which are necessary for programming the infected host cell to synthesize virus-specific macromolecules required for the production of viral progeny, indicating that vAcRev-1 likely cannot produceany viral progeny by itself. The 10 essential genes are *ac46* (*odv-e66*)^31^, *ac50* (*lef8*)^32^, *ac51* (*dnaj domain protein*)^33^, *ac53*^34^, *ac53a*^35^, *ac54* (*vp1054*)^36^, *ac62* (*lef9*)^37^, *ac65* (*dna polymerase*)^38^, *ac66*^39^ and *ac67* (*lef3*)^40^.

Except for several ORFs which have a single nucleotide inserted or substituted, vAcRev-2 and vAc^∆P35^ share over 99% nucleotide sequence or amino acid identities between the corresponding ORFs. Since vAc^∆P35^ cannot replicate efficiently in Sf9, Se301 and SpLi-221 cell lines due to apoptosis, it is expected that vAcRev-2 would induce apoptosis and have aborted replication in these cell lines as well.

As single enzyme *Bsu36*I loci were found in both of the genome of vAcRev-1 and vAcRev-2, vAcRev DNA was linearized by *Bsu*36I digestion and then analyzed by pulsed field gel electrophoresis. The electrophoretogram exhibited two enzyme-digested fragments consistent with the expected sizes of vAcRev-1 and vAcRev-2 (Fig. 3B). This result confirmed that vAcRev is composed of a mixture of vAcRev-1 and vAcRev-2. The intensity of the two molar fragments is comparable, indicating that the molar ratio of vAcRev-1 and vAcRev-2 was approximately 1:1.

The above genomic analyses, together with the plaque morphology results, suggested that vAcRev-1 and vAcRev-2 cannot replicate individually in Sf9, Se301 and SpLi-221 cell lines due to the lack of essential genes and lack of ability to inhibit apoptosis, respectively. Thus, we argue that vAcRev-1 and vAcRev-2 exist in a mutualistic relationship where in vAcRev-1 provides an anti-apoptotic gene (the truncated *Se-iap3* in vAcRev-1, which we have named vAcRev*-iap3*), while vAcRev-2 provides the essential genes which vAcRev-1 lacks.

### RNAi of *vAcRev-iap3* results in apoptosis of vAcRev-infected Sf9 cells

**Figure 5:**
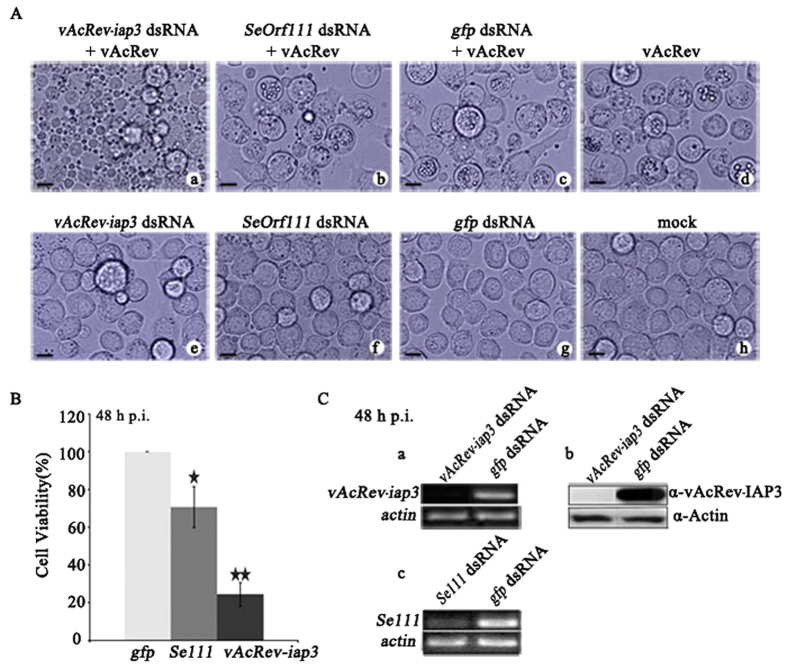
Effect of *vAcRev-iap3* dsRNA on vAcRev-infected SpLi-221 cells. (**A**) SpLi-221 cells were transfected with 2 μg dsRNA corresponding to *vAcRev-iap3*, *SeOrf111* or *gfp*. At 24 h p.t., the transfected-cells were infected with vAcRev and then observed at 48 h p.i. (**B**) Cell viability of the treated cells at 48 h p.i. The mean values and standard deviation of three independent experiments are shown. All data were analyzed by *t*-test using independent samples. A *P*-value of less than 0.01 was considered very significant. *P ≤ 0.01. (**C**) Levels of mRNA and protein of *vAcRev-iap3* were determined at 48 h p.i. by RT-PCR (a) or immunoblotting (b), respectively. Transcript levels were also determined for *Se111* (c).

To determine whether the newly integrated SeMNPV genes are critical to the replication of vAcRev, RNAi was carried out. The truncated version of *Se-lef8* integrated in vAcRev1 is a short C-terminal region of the *Se-lef8* ORF and was assumed to not produce a functional protein. Thus we focused on *Se-iap3* and *Se-orf111*. SpLi-221 cells were transfected with dsRNAs of *vAcRev-iap3* or *Se111*, and 24 h later the cells were infected with vAcRev at a multiplicity of infection (MOI) of 20 TCID_50_/cell (50% tissue culture infective dose per cell). At 18 h p.i., plasma membrane blebbing (a marker of apoptosis) was first observed in a small proportion of cells treated with *vAcRev-iap3* dsRNA, and the number of cells showing apoptosis increased from then on. By 48 h p.i., most of the *Se-iap3* dsRNA-treated, vAcRev-infected cells had undergone apoptosis, while cells treated with *Se-111* or *gfp* dsRNA and infected with vAcRev did not exhibit apoptosis, but instead showed typical signs of infection including PIB formation (Fig. 5A). Trypan blue staining assay showed that the relative survival rates (compared with *gfp* dsRNA-treated vAcRev-infected SpLi-221 cells) of *vAcRev-iap3* and *Se111* dsRNA-treated vAcRev-infected cells were 24.4% and 70.6%, respectively (Fig. 5B). DsRNA-treated cells without vAcRev infection did not undergo apoptosis (Fig. 5A). The silencing of vAcRev-IAP3 expression was confirmed by RT-PCR and western blot analysis (Fig. 5C). These results suggest that vAcRev-IAP3 acts as the primary helper to extend the host range of vAcRev, and *Se111* does not appear to be necessary for replication of vAcRev but might increase the survival rate of infected cells.

### *vAcRev-iap3* is a truncated version of *Se-iap3* and is expressed at early/late infection stages

The *iap3* gene cassette of vAcRev was subsequently analyzed and compared with its counterpart from SeMNPV. The vAcRev-*iap3* ORF is 897 bp in length and encodes a polypeptide of 298 amino acids, with a predicted molecular mass of 34.2 kDa (Figure S1 in the supplemental materials). Amino acid sequence alignment showed that vAcRev-IAP3 is a truncated version of Se-IAP3 that is lacking the first 16 amino acids at the N-terminus. vAcRev-IAP3 still contains the two BIR domains at the N-terminus and the RING finger motif at the C-terminus of Se-IAP3, which are both typical motifs for IAP proteins. The BIR motifs of baculovirus, vertebrate, and invertebrate exhibit several rigorously conserved residues, including three cysteines and a histidine that coordinate an atom of zinc in the center of a hydrophobic core. However, the second conserved cysteine residue of the SeMNPV-IAP3 BIR2 motif (CX_2_CX_16_HX_6_C) is replaced with a serine residue and this replacement is also found in vAcRev-IAP3 (Figure S2 in the supplemental materials).

**Figure 6:**
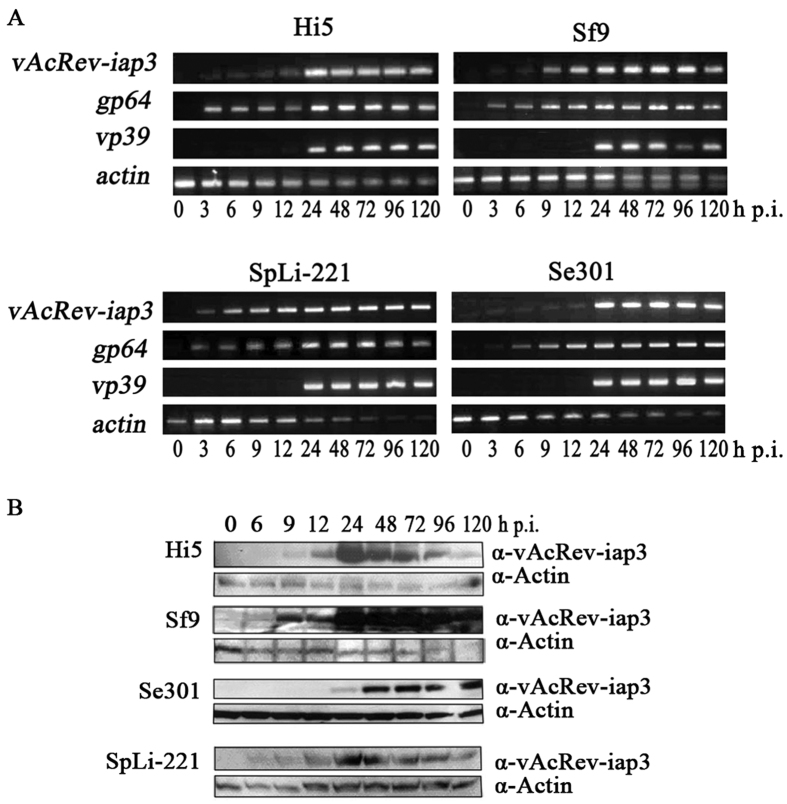
*vAcRev-iap3* gene expression analysis in vAcRev-infected cells. (**A**) RT-PCR analysis of the temporal transcripts of *vAcRev-iap3*. Total RNAs were prepared from vAcRev-infected Hi5, Sf9, Se301 and SpLi-221 cells and were subjected to RT-PCR analysis using primers that amplify *vAcRev-iap3*, and *gp64*, *vp39*, host *actin* gene was amplified as a control. (**B**) Time course of vAcRev-IAP3 expression during vAcRev infection. 50 μg of total protein from vAcRev-infected cells at the indicated time points were separated on 12% SDS-polyacrylamide gel, and then analyzed by western blotting with mouse vAcRev-IAP3 or actin monoclonal antibody, visualized by goat anti-mouse IgG antibody conjugated with horseradish peroxidase and ECL. Time points p.i. hours are indicated above the lanes.

The canonical baculovirus promoter motifs for early genes (TATA box followed by CAGT) and late genes (TAAG) are present upstream of the predicted translation start codon of SeMNPV *iap3*. However, neither was found within 500 nt upstream from the predicted translation start codon of vAcRev-*iap3*. Similar to SeMNPV *iap3*, a consensus polyadenylation signal (AATAAA motif) is located 14 nucleotides downstream from the stop codon of vAcRev-*iap3* (Fig. S1). Thus, the time-course of vAcRev-*iap3* transcription and expression during vAcRev infection was investigated. RT-PCR showed that vAcRev-*iap3* transcripts could be detected as early as 3 h p.i. in vAcRev-infected Hi5, Sf9, Se301 and SpLi-221 cells (Fig. 6A), suggesting that vAcRev-*iap3* might be an early gene that is transcribed before viral DNA replication. The transcripts continued to be detectable up to 96 h p.i. By using a prepared anti-vAcRev-IAP3 monoclonal antibody, a major immunoreactive band of approximately 36 kDa was first detected at 6 h p.i. in vAcRev-infected Sf9 and SpLi-221 cells, 9 h p.i. in vAcRev-infected Hi5, and 24 h p.i. in Se301 cells, and the protein amount increased gradually through the late phase of infection (Fig. 6B). The size of the protein is consistent with the predicted 34.2 kDa of the vAcRev-*iap3* gene, suggesting that no major post-translational modification occurs.

### Expression of SeIAP3 prevents apoptosis and rescues vAc^∆P35^ replication

**Figure 7:**
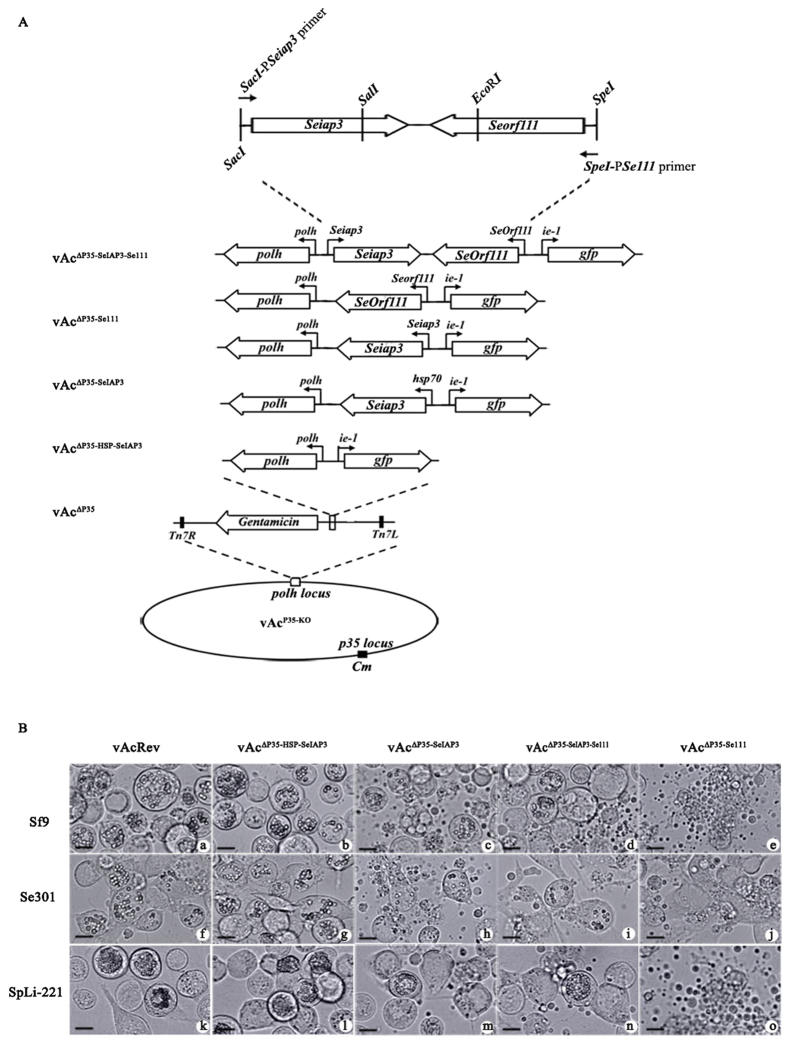
Replication of constructed viruses carrying SeIAP3, Se111, or both genes. (**A**) Schematic diagram of the constructed viruses. (**B**) Light micrographs of insect cells infected with indicated viruses at 72 h p.i.

To further study the mechanism of the vAcRev host range expansion, four AcMNPV recombinants were constructed: vAc^∆P35-HSP-SeIAP3^ (lacking P35 but bearing SeIAP3 which was under the control of *hsp70* promoter), vAc^∆P35-SeIAP3-Se111^ (lacking P35 but bearing SeIAP3 and Se111 expression cassettes), vAc^∆P35-SeIAP3^ (lacking P35 but bearing SeIAP3 expression cassettes) and vAc^∆P35-Se111^ (lacking P35 but bearing Se111 expression cassette) (Fig. 7A).

The three vAc^∆P35^ non-permissive cell lines (Se301, Sf9 and SpLi-221 cells) infected with vAc^∆P35-SeIAP3^ and vAc^∆P35-SeIAP3-Se111^ exhibited a mixed phenotype, where some infected cells underwent apoptosis but other cells produced PIBs. Infection withvAc^∆P35-Se111^ caused widespread apoptosis, which was comparable to vAc^∆P35^. However, apoptosis was totally inhibited and PIBs were formed in vAc^∆P35-HSP-SeIAP3^-infectedcells (Fig. 7B), in which Se-IAP3 was expressed from the strong constitutive *hsp70* promoter^41^. These results suggested strongly that Se-IAP3 is a functional apoptosis inhibitor. Interestingly, our previous research showed that overexpression of Splt-P49 cannot support vAc^∆P35^ replication in SpLi-221 cells, even though the overexpression blocks virus-induced apoptosis^27^. Our results suggested that Se-IAP3 might facilitate the vAc^∆P35^ replication.

## Discussion

In the present study, by co-transfecting Sf9 cells with an AcMNPV mutant which has a deletion of the anti-apoptotic gene *p35* (vAc^∆P35^) and cosmid 22 which represents a fragment of SeMNPV, a host range expanded recombinant virus strain (vAcRev) was generated. Host specificity of vAcRev was analyzed in four insect cell lines and three insect larvae. Substantial accumulation of BVs and PIBs indicates productive infection of vAcRev in Hi5, Sf9, SpLi-221 and Se301 cell lines. *T. ni* and *S. exigua* larvae are sensitive to vAcRev by oral inoculation. Moreover, vAcRev can kill *S. litura* larvae by hemocoelic injection of BV while the *p35*-null mutant cannot. These *in vivo* and *in vitro* assays indicate that vAcRev has a broader host range than its parental viruses vAc^∆P35^ and SeMNPV.

Restriction endonuclease patterns showed that the genetic material of vAcRev is mainly derived from vAc^∆P35^. Genomic sequencing and comparisons revealed that vAcRev is a mixture of two virus genotypes (vAcRev-1 and vAcRev-2). The main distinction between the vAcRev-1 and vAcRev-2 genomes is a region where 24.4 kb nucleotide sequence containing 26 ORFs (*Ac43*~*Ac68*) is replaced by a 4.0 kb nucleotide sequence containing three ORFs of SeMNPV (vAcRev*-iap3*, intact *Se111* and truncated *Se-lef8*). Since the replaced region contains 10 essential genes, vAcRev-1 presumably cannot replicate on its own in cells, but it possesses a potential anti-apoptosis gene (vAcRev*-iap3*) which can resist the apoptosis response induced by vAcRev infection. In addition, as vAcRev-2 has a similar gene content and a similar gene arrangement to the genome of vAc^∆P35^, vAcRev-2 alone also presumably cannot replicate in Sf9, Se301 and SpLi-221 cells, but it provides the gene products of *Ac43~Ac68*, especially the 10 essential genes, to support vAcRev replication. Thus we propose that vAcRev-1 and vAcRev-2 are dependent on each other to replicate in Sf9, Se301 and SpLi-221 cells.

Recombination plays an important role in the generation of variability between virus strains and can produce recombinants with novel characteristics^42^. Recombination between two baculovirus genomes or between a baculovirus genome and cosmid/plasmid DNA representing a fragment of a baculovirus genome sometimes results in chimeric viruses with expanded host ranges^6^,^15^,^16^,^43^. However, to the best of our knowledge, the host range of the previously reported recombinant viruses is limited to insects that are hosts for one of the parental viruses. Thus, vAcRev is the first reported recombinant baculovirus that is derived from *in vitro* recombination in which the host range has expanded to a host that is non-permissive for both parental viruses, namely *S. litura* larvae and SpLi-221 cells.

Most baculovirus *iap3* genes tested to date have anti-apoptotic ability^44^. When vAcRev*-iap3* expression was knocked down by vAcRev*-iap3* dsRNA, vAcRev infection induced apoptosis, indicating that vAcRev*-iap3* is the responsible for suppressing apoptosis in vAcRev. To further investigate the role of *iap3* in the vAcRev host range expansion to SpLi-221 cells, we constructed a series of recombinant viruses in which *Se-iap3* was under the control of different promoters. Apoptosis was not suppressed completely in vAc^∆P35-SeIAP3^-infected cells in which *Se-iap3* expression was under the control of its native promoter, while apoptosis could be totally inhibited in vAc^∆P35-HSP-SeIAP3^-infected cells in which *Se-iap3* was expressed under the control of the constitutive *hsp70* promoter. Moreover, occlusion bodies formed in the infected cells, indicating that infection could progress into the very late phase. Considering that both P35 and P49 are able to block apoptosis but fail to rescue viral replication in these cases, our results suggested that that either 1) apoptosis is the only block to AcMNPV infection in *S. litura* cells which is not consistent with our earlier results^27^, or 2) Se-IAP3 has another function that promotes viral replication other than inhibition of apoptosis.

Over-expression of SeIAP3 may have the same effects in vAcRev-infected cells. Although vAcRev-IAP3 is a truncated Se-IAP3 lacking the first 16 amino acid at the N-terminus, the typical IAP functional motifs, including two BIR motifs and a Ring-finger motif, remain. Thus we conclude that vAcRev-IAP3 acts similarly to SeIAP3. Interestingly, unlike the promoter region of *Se-iap3* in which there are the consensus sequences of baculovirus promoter CAGT or TAAG motifs, a TATA box followed by a poly T fragment exists in the promoter region of vAcRev*-iap3.* We speculate that this region might contain a strong promoter to drive the expression of vAcRev*-iap3*.

There is a rapidly growing body of evidence revealing that IAPs are more than just inhibitors of apoptosis, but that they also play an important role in adaptive response to cellular stress, in cell proliferation, differentiation, signaling, motility and in immune response. Our findings that a viral IAP is involved in virus replication in a non-permissive host may open exciting perspectives for the future genetic engineering of baculoviruses.

## Experimental procedures

### Cells, larvae, and viruses

The Sf9 cell strain is a clonal derivative of the Sf21 cell line, derived from the fall armyworm (*S. frugiperda*)^45^. The Se301 cell strain is derived from the beet armyworm (*S. exigua*)^46^. The cell line TUAT-SpLi221 (SpLi-221) is derived from the tobacco cutworm (*S. litura*)^15^. The BTI-Tn-5B1-4 (Hi5) cell strain is derived from the cabbage looper (*T. ni*)^15^,^36^. These cells were maintained at 27 °C in TNM-FH medium (Invitrogen Life Technologies) supplemented with 10% fetal bovine serum (FBS), penicillin (100 μg/ml), and streptomycin (30 μg/ml). The *S. exigua*, *S. litura*, and *T. ni* larvae were reared on artificial diet at 27 °C under a 14/10 h light/dark cycle and constant humidity (60%)^47^.

The AcMNPV E2 strain, which was referred to as vAc^WT^ in this paper, was propagated in *T. ni* larvae, Sf9 cells, or Hi5 cells. The SeMNPV US1 strain^48^,^49^ was propagated in *S. exigua* larvae or Se301 cells. The SpltNPV G2 strain^50^ was propagated in *S. litura* larvae or SpLi-221 cells. vAc^P35-KO^ is a *p35*-null AcMNPV bacmid^27^. To facilitate the examination of viral infection, the AcMNPV *polyhedrin* (*ph*) and *enhanced green fluorescence protein* (*gfp*) genes were inserted into the *ph* locus of vAc^∆P35-KO^ via site-specific transposition as previously described^51^, and the resulting virus was designated vAc^∆P35^. PIBs were propagated and purified as previously described^42^. BVs were harvested from the infected larva hemolymph at 3 d p.i.^52^. The BV titers were determined by TCID_50_ end point dilution assay in corresponding cells^52^.

The SeMNPV cosmid library, which represents the whole viral genome and consists of cosmid 24, cosmid 17, cosmid 32, cosmid 7 and cosmid 22^28^, was provided by Prof. Just M. Vlak (Department of Virology, Wageningen University, the Netherlands).

The cosmid and bacmid DNAs were extracted using a QIAGEN Large-Construct Kit and were quantified by optical density.

### Cotransfection of vAc^∆P35^ and the SeMNPV cosmid(s) into Sf9 cells

Sf9 cells (1 × 10^6^) were cotransfected with 1 μg vAc^∆P35^ and SeMNPV cosmid(s) DNA either together or individually, by using Cellfectin liposome reagent (Invitrogen Life Technologies). At 5–10 days p.t., cells were examined by light microscopy. The PIB-positive recombinant virus was plaque-purified as described previously^53^.

### One-step virus growth curve

The titers of vAcRev were determined generally in Hi5 cells. To compare infectious BV production in different cells, SpLi-221, Se301, Hi5 and Sf9 cells (5.0 × 10^5^ cells for each cell line) were infected with vAcRev at a MOI of 20 TCID_50_/cell, respectively. The supernatants were collected at different time points, and were titered in the corresponding cells. Time zero was defined as the time when the inoculum was added to the cells. Five photographs of the infected cells were randomly taken at 48 and 72 h p.i., and the percentages of cells containing PIBs were calculated.

### Bioassays

Bioassays were performed on newly molted third-instar larvae of *T. ni* and *S. exigua* by feeding with a small piece of diet containing 1 μl PIB solution or water (control). Due to the different infectivity to larva, various concentrations were applied. For AcMNPV, vAcRev or vAc^∆P35^ in *T. ni* larvae, 9 × 10^6^, 3 × 10^6^, 9 × 10^5^, 3 × 10^5^, and 9 × 10^4^ PIBs/ml were chosen. For AcMNPV, vAcRev, vAc^∆P35^ in *S. exigua* larvae, 9 × 10^7^, 3 × 10^7^, 9 × 10^6^, 3 × 10^6^, and 9 × 10^5^ PIBs/ml were chosen. For SeMNPV in *S. exigua* larvae, 3 × 10^6^, 9 × 10^5^, 3 × 10^5^, 9 × 10^4^, and 3 × 10^4^ PIBs/ml were chosen. Twenty-four larvae were used per treatment.

Because of the lack of oral infectivity of vAcRev PIBs to *S. litura*, its BV infectivity was determined by hemocoelic inoculation. Middle fourth-instar *S. litura* larvae were injected with 5 μl of vAcRev (5 × 10^4^, 1 × 10^4^, 5 × 10^3^, 1 × 10^3^ and 5 × 10^2^ TCID_50_/ml titrated in SpLi-221 cells). SpltNPV BVs (1 × 10^4^, 1 × 10^3^, 1 × 10^2^, 1 × 10^1^ and 1 × 10^0^ TCID_50_/ml) were used as a positive control, and vAc^∆P35^ BVs (5 × 10^5^, 1 × 10^5^, 5 × 10^4^, 1 × 10^4^ and 5 × 10^3^ TCID_50_/ml titrated in Hi5 cells) were as negative control. TNM-FH medium as were used as a blank control.

Three replicates were performed for each trial. Larval death was monitored daily until larvae died or pupated. Data was conducted using the SPSS data processing software.

### Restriction endonuclease analysis and genome sequencing

Viral DNAs were extracted from PIBs as described previously^42^. The DNAs were analyzed by pulsed-field gel electrophoresis or digested with restriction endonucleases as described elsewhere^54^.

The vAcRev genome was sequenced by using 454 pyrosequencing^55^. Bioinformatics pipelines were used to assemble contigs from the sequence data production^56^. The draft assembly was followed by a labor-intensive finishing phase where the assembled sequences were improved using targeted sequencing to resolve misassembled regions, close sequence gaps, and improve coverage and accuracy in sparsely covered regions of the genome.

### Generation of dsRNAs and RNA interference (RNAi)

dsRNAs were prepared as described previously^57^,^58^. Briefly, a 286-bp fragment of vAcRev-*iap*3 and a 477-bp fragment of *gfp* were PCR-amplified by using vAcRev DNA as template. The PCR primers were designed to contain a 5′ T7 RNA polymerase binding site (TAATACGACTCACTATAGG) followed by sequences specific for target genes. The sequences of the primer pairs are as follows. For *iap3*: T7-vAcRev-iap3-U (CGGGATCC*TAATACGACTCACTATAGG*AATTGGAGAGAGGGCGACGATC, *Bam*HI site is underlined) and T7-vAcRev-iap3-D (GGAATTC*TAATACGACTCACTATAGG*AATCACATGACCGCACGGCA, *Eco*RI site is underlined). For *gfp*: T7-gfp-U (CGGGATCC*TAATACGACTCACTATAGG*GTGTTCAATGCTTTTCAAGATAC, *Bam*HI site is underlined) and T7-gfp-D (GGAATTC*TAATACGACTCACTATAGG*CTGTTACAAACTCAAGAAGGACC, *Eco*RI site is underlined). After purification with a High Pure PCR Purification Kit (Roche Molecular Biochemicals), the PCR products were used as templates to produce dsRNA using an Ampliscribe^TM^ T7*-Flash*^TM^ Transcription Kit (EPICENTER). RNase-free DNase was added to digest the DNA templates. After incubation at 95 °C for 2 min and slow cooling to room temperature, the annealed dsRNA were purified by using an RNeasy Mini Kit (QIAGEN). The concentrations of dsRNA were determined with a spectrophotometer. The resulting dsRNAs were stored at −70 °C until use.

SpLi-221, Sf9 or Se301 cells were mock-transfected or transfected with 2 μg of *vAcRev-iap3* or *gfp* dsRNA using Trans Messenger Transfection Reagent (Qiagen). Twenty-four hour later, the transfected cells were infected with vAcRev at an MOI of 10 TCID_50_/cell. The cells were observed for the occurrence of apoptosis with a light microscope.

### Cell viability assay

Viable cell numbers were assessed by using trypan blue staining assay as previously described^59^.

### Transcriptional analysis of vAcRev-*iap3*

Hi5, Sf9, Se301, and SpLi-221 cells were infected with vAcRev at an MOI of 20 and were harvested at different time points (p.i.), respectively. Time zero was defined as the time when the inoculum was added to the cells. Total cellular RNA was extracted as mentioned above. RT-PCR was conducted using a RNA PCR Kit (TaKaRa, Ver.3.0) employing 1 μg of total RNA as template. The PCR products were analyzed using a 1% agarose gel.

### Antibody preparation and immunoblot analysis

Two peptides corresponding to amino acids 79 to 275 and 1 to 313 of SeMNPV IAP3 were synthesized by Abmart and were used to generate polyclonal antibodies in three mice. The monoclonal antibodies of SeMNPV IAP3 were screened by bone marrow hybridoma fusion technology, and the spleen cells of mice which had the best immune response were fused with myeloma cells (SP2/0).

Western blotting was performed by standard procedures as previously described^60^. Briefly, the infected-cells were harvested and lysed at the indicated time points. The mouse monoclonal anti-vAcRev-IAP3 antibody (1:200) or a mouse monoclonal anti-actin antibody (1:2000; Abmart) were used as the primary antibodies, and horseradish peroxidase-conjugated anti-mouse IgG (1:5000; GE Healthcare) as a secondary antibody. The blots were visualized using enhanced chemiluminescence reagent (ECL Advance Western Blotting Detection Kit; GE Healthcare).

### Construction of recombinants

Using SeMNPV DNA as template, a 1.6-kbp fragment containing SeMNPV *iap3-orf111* was PCR amplified with primers *Spe*I-P*Se111* (5′-ACTAGTCATTTGTGTAGCACGCATCG-3′) and *Sac*I-P*Seiap3* (5′-GAGCTCGTACACAATGTTTGCTTTCG-3′) (*Spe*I and *Sac*I sites are underlined, respectively). The PCR product was cloned into pMD18-T (Invitrogen Life Technologies) to generate plasmid pT-SeIAP3-Se111. Using pT-SeIAP3-Se111 as a template, the SeMNPV *iap3* orf plus its own poly(A) signals was PCR amplified with primers *Bam*HI-*Seiap*3-5′ (5′-GGATCC**ATG**CAGGTGAACAGCGATGAATC-3′) and *Spe*I-*Seiap3*-polyA (5′-ACTAGTGAAGCAGCGGAGACGAGTTG-3′) (*Bam*HI and *Spe*I sites are underlined, respectively). The PCR product was cloned into pMD18-T to generate plasmid pT-SeIAP3. By using plasmid phsp-IAP^59^ as template, the *hsp70* promoter was PCR amplified with primers *Hsp70-Sac*I (5′-GAGCTCCTAGAATCCCAAAACAAACT-3′) and *Hsp70-Bam*HI (5′-GGATCCTTCAGAGTTCTCTTCTTGTA-3′) (*Sac*I and *Bam*HI sites are underlined, respectively). The PCR product was cloned into pMD18-T to generate plasmid pT-P*hsp70*. pT-P*hsp70* was digested with *Bam*HI and *Sac*I, and the digested product was ligated into pT-SeIAP3 to generate plasmid pT-*hsp70*-SeIAP3. pT-*hsp70*-SeIAP3 was digested with *Spe*I and *Sac*I, and the digested product was ligated into pFB1-PH-GFP to generate the donor plasmid pFB1-PH-*hsp70*-SeIAP3-GFP. pT-SeIAP3-Se111 was separately digested with *Spe*I and *Sac*I, *Sac*I and *Eco*RI, *Spe*I and *Sal*I to generate three fragments containing *iap3-orf111*, *iap3*, and *orf111* gene cassettes, respectively. The digested products were ligated into pFB1-PH-GFP^51^, to generate the donor plasmids pFB1-PH-SeIAP3-Se111-GFP, pFB1-PH-SeIAP3-GFP, and pFB1-PH-Se111-GFP. The different genes were inserted into the *ph* locus of vAc^P35-KO^ via site-specific transposon as previous described^51^, to generate vAc^∆P35-HSP-SeIAP3^, vAc^∆P35-SeIAP3-Se111^, vAc^∆P35-SeIAP3^ and vAc^∆P35-Se111^, respectively.

## Additional Information

**How to cite this article**: Wu, C. *et al*. Generating a host range-expanded recombinant baculovirus. *Sci. Rep.* **6**, 28072; doi: 10.1038/srep28072 (2016).

## Supplementary Material

Supplementary Information

## Figures and Tables

**Table 1:** Dose-mortality response of insect larvae infected with different viruses.

Host and viruses	Conc.set^1^	LD_50_^2^	95% fiducial limits^3^	Slope
			(Lower–upper)	
*T. ni*(o)				
vAcRev	1	82a	(64.4–104.4)	1.2263
AcMNPV	1	111.1ab	(87.1–141.5)	1.2037
vAc^∆P35^	1	141.1b	(109.0–182.7)	1.1267
*S. exigua*(o)				
vAcRev	2	5183.8c	(4003.4–6712.4)	1.1328
AcMNPV	2	4219.4c	(3528.1**–**5046.1)	1.9109
vAc^∆P35^	2	6214.2c	(4576.8**–**8437.3)	0.9082
SeMNPV	3	166.0d	(134.4–205.2)	1.4350
*S. litura*^(i)^				
vAcRev	4	3.026e	(2.262–4.047)	0.9705
vAc^∆P35^	5	–	–	–
SpltNPV	6	0.011f	(0.008–0.017)	0.8731

^(o)^Orally inoculated; ^(i)^intrahemocoelically inoculated.

^1^Concentration sets were selected as: set 1: 9 × 10^6^, 3 × 10^6^, 9 × 10^5^, 3 × 10^5^, and 9 × 10^4^ PIBs/ml; set 2: 9 × 10^7^, 3 × 10^7^, 9 × 10^6^, 3 × 10^6^, and 9 × 10^5^ PIBs/ml; set 3: 3 × 10^6^, 9 × 10^5^, 3 × 10^5^, 9 × 10^4^,and 3 × 10^4^ PIBs/ml; set 4: 5 × 10^4^, 1 × 10^4^, 5 × 10^3^, 1 × 10^3^, and 5 × 10^2^ TCID50/ml; set 5: 5 × 10^6^, 5 × 10^5^, 5 × 10^4^, 5 × 10^3^ and 5 × 10^2^ TCID_50_/ml; set 6: 1 × 10^4^, 1 × 10^3^, 1 × 10^2^, 1 × 10^1^ and 1 × 10^0^ TCID_50_/ml.

^2&3^For orally infected larvae, LD_50_ = PIBs/larvae; for intrahemocoelically-infected larvae, LD_50_ = TCID_50_/larvae. The mean values and standard deviation of three independent experiments are shown. All data were analyzed by *t*-test using independent samples. A *P*-value of less than 0.05 was considered significant. The same letters (a~f) behind LD_50_ indicate that the LD_50_ values are not significantly different.
