# Supplementary material for: Generating a host range-expanded recombinant baculovirus
Source: Sci Rep. 2016 Jun 20;6:28072. doi: 10.1038/srep28072 (PMC4913269; doi:10.1038/srep28072)
Supplement: Supplementary Information [file srep28072-s1.doc]

**Title:** Generating a host range-expanded recombinant baculovirus

**Running title:**

A host range-expanded recombinant baculovirus

**Authors:**

Chunfeng Wu^1,3^, Zihao Deng^1^, Zhao Long^1^, Yi Cai^1^, Zhongfu Ying^1^, Hanqi Yin^1^, Meijin Yuan^1^, Rollie J. Clem^2^, Kai Yang^1^,*, Yi Pang^1^

**Author Affiliation:**

1, State Key Laboratory of Biocontrol, SunYat-sen University, Guangzhou 510275, China

2, Division of Biology, Kansas State University, Manhattan, KS66506,

3, Liuzhou People's Hospital, Liuzhou 545006, China

**Corresponding Author:**

*Kai Yang. State Key Laboratory of Biocontrol, SunYat-senUniversity, Guangzhou 510275, China

**Phone:** +86(0)20 84036809, **Fax:** +86(0)20 84037472,

**E-mail:** yangkai@mail.sysu.edu.cn


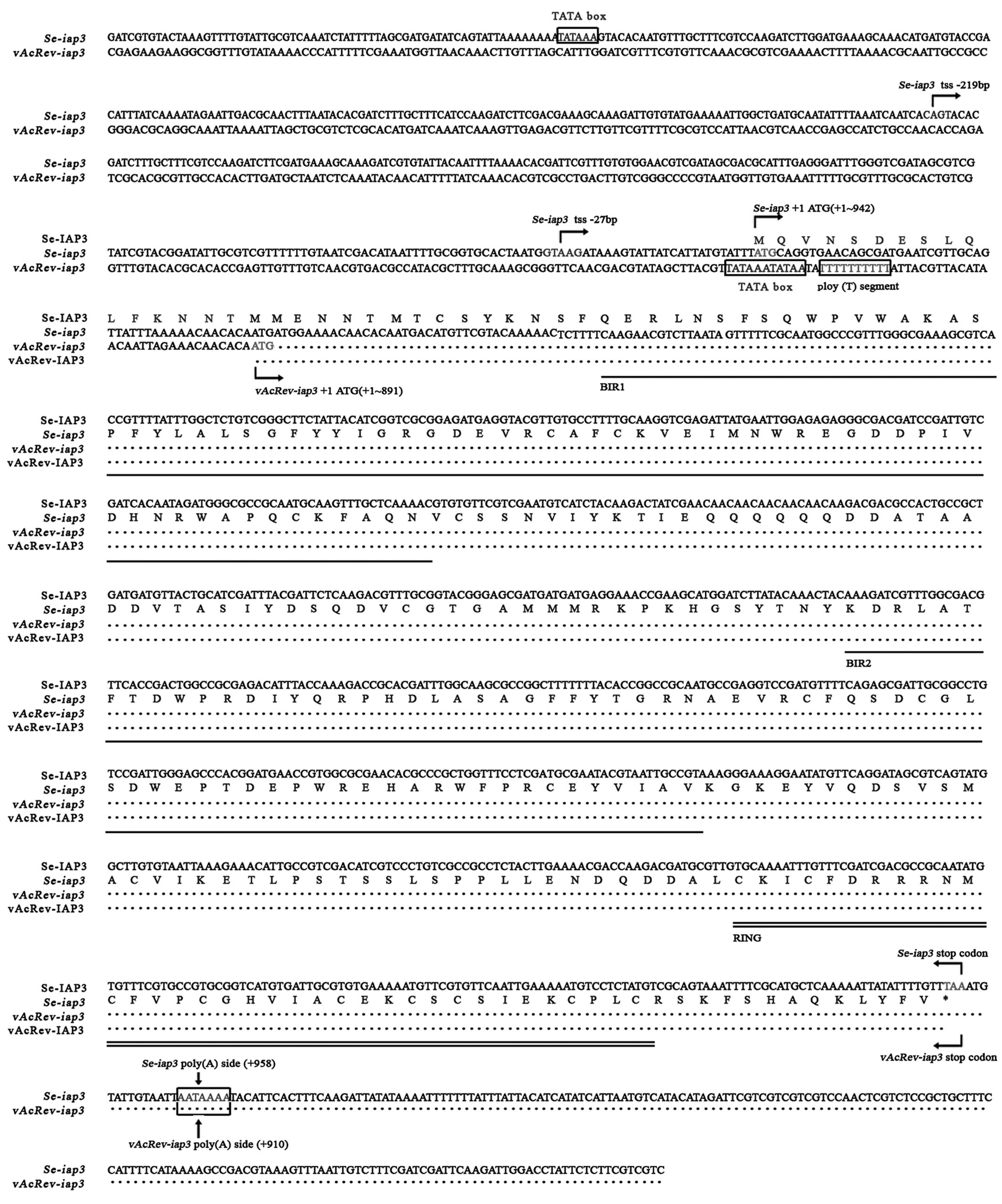


Fig. S1. Nucleotide and deduced amino acid sequence of vAcRev-IAP3 and Se-IAP3. The location of translation start codon (ATG), stop codon (TAA), early (CATG following TATA box) and late (TAAG) promoter motifs, along with a poly T region, are indicated. The two BIRs are underlined and the RING finger motif is doubly underlined.


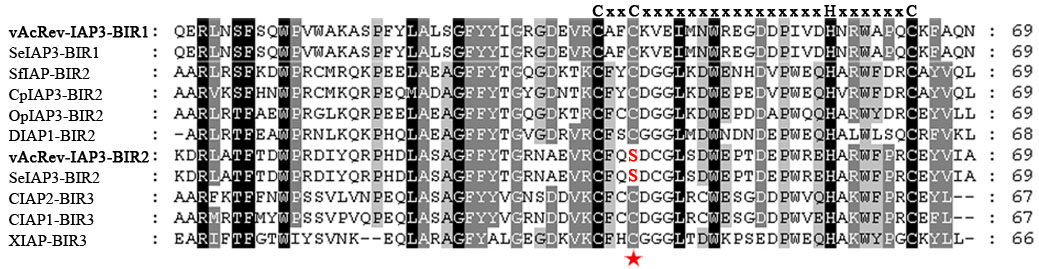


Fig. S2. Alignment and structure analysis of BIR domains in vAcRev-IAP3. Alignment by the ClustalW method of the BIR domains of baculovirus, vertebrate, and invertebrate IAPs. Amino acid residues that are conserved in sequences are shaded in dark and similar amino acids are shaded in grey. It was found that there was serine substituted in place of the second cysteine at the BIR signature sequence CX_2_CX_16_HX_6_C (X denotes any amino acid) in the vAcRev-IAP3 and Se-IAP3 BIR2 domains, which are marked in red.

# Table. S1. Genomic comparison of AcMNPV, vAcRev-1 and vAcRev-2

| **AcMNPV** | | | | | **vAcRev-1** | | | | | | **vAcRev-2** | | | | | |
| --- | --- | --- | --- | --- | --- | --- | --- | --- | --- | --- | --- | --- | --- | --- | --- | --- |
| **ORF^1^** | **Name^2^** | **Position^3^** | **nt** | **aa** | **Name^2^** | **Position^3^** | **nt** | **aa** | **Identity^4^** | | **Name^2^** | **Position^3^** | **nt** | **aa** | **Identity^4^** | |
|  |  |  |  |  |  |  |  |  | **nt** | **aa** |  |  |  |  | **nt** | **aa** |
| hr1 |  | Join  (133883..133894,  1..445) |  |  |  | Join  (118571..118582,  1..445) |  |  |  |  |  | Join  (138980..138991,  1..445) |  |  |  |  |
| 1 | *ptp* | 503→1009 | 507 | 168 | *** | *** | * | * | - | - | *** | * | * | * | - | - |
| 2 | *bro* | 1041←2027 | 987 | 328 | *** | 1006←2028 | 1023 | 340 | 0.965 | 0.924 | *** | # | # | # | # | # |
| 3 | *ctx* | 2084←2245 | 162 | 53 | *** | 2085←2246 | * | * | - | - | *** | # | * | * | - | - |
| 4 | *ac4* | 2295←2750 | 456 | 151 | *** | 2296←2751 | * | * | - | - | *** | # | * | * | - | - |
| 5 | *ac5* | 2779→3108 | 330 | 109 | *** | 2780→3109 | * | * | - | - | *** | # | * | * | - | - |
| 6 | *lef-2* | 3089→3721 | 633 | 210 | *** | 3090→3722 | * | * | - | - | *** | # | * | * | - | - |
| 7 | *orf603* | 3759←4364 | 606 | 201 | *** | 3760←4365 | * | * | - | - | *** | # | * | * | - | - |
|  |  |  |  |  | ***kanR*** | **4777←5592** | **816** | **271** |  |  | # | # | # | # |  |  |
|  |  |  |  |  | ***gfp*** | **6553←7269** | **717** | **238** |  |  | # | # | # | # |  |  |
| 8 | *ph* | 4520→5257 | 738 | 245 | *** | 8072→8809 | * | * | - | - | *** | # | * | * | - | - |
| 9 | *vp78/* | 5287←6918 | 1632 | 543 | *** | 8839←10470 | * | * | - | - | *** | # | * | * | - | - |
| 10 | *pk-1* | 6917→7735 | 819 | 272 | *** | 10469→11287 | * | * | 0.998 | 0.982 | *** | # | * | * | # | # |
| **hr1a** |  | 7747-7864 | 118 |  |  | 11299-11416 | * |  | - |  |  | # | * |  | - |  |
| 11 | *ac11* | 7899←8921 | 1023 | 340 | *** | 11451←12473 | * | * | - | - | *** | # | * | * | - | - |
| 12 | *ac12* | 8958→9611 | 654 | 217 | *** | 12510→13163 | * | * | 0.998 | 0.995 | *** | # | * | * | # | # |
| 13 | *ac13* | 9638←10621 | 984 | 327 | *** | 13190←14173 | * | * | - | - | *** | # | * | * | - | - |
| 14 | *lef-1* | 10513←11313 | 801 | 266 | *** | 14065←14865 | * | * | - | - | *** | # | * | * | - | - |
| 15 | *egt* | 11426→12946 | 1521 | 505 | *** | 14978→16495 | 1518 | 505 | 0.997 | 0.998 | *** | # | # | # | # | # |
| 16 | *bv/odv-e26* | 13092→13769 | 678 | 225 | *** | 16644→17321 | * | * | 0.999 | 0.996 | *** | # | * | * | # | # |
| 17 | *ac17* | 13738→14232 | 495 | 164 | *** | 17290→17919 | 630 | 209 | 0.783 | 0.781 | *** | # | # | # | # | # |
| 18 | *ac18* | 14398←15459 | 1062 | 353 | *** | 17949←19010 | * | * | 0.999 | - | *** | # | * | * | # | - |
| 19 | *ac19* | 15461→15787 | 327 | 108 | *** | 19012→19338 | * | * | - | - | *** | # | * | * | - | - |
| 20 | *ac20* | 16013←16222 | 210 | 69 | ***ac20/21*** | **19564←20817** | **1254** | **417** |  |  | # | # | # | # |  |  |
| 21 | *ac 21* | 16305←17264 | 960 | 319 |  |  |  |  |  |  |  |  |  |  |  |  |
| 22 | *pif-2* | 17301→18449 | 1149 | 382 | *** | 20854→22002 | * | * | - | - | *** | # | * | * | - | - |
| 23 | *env-prot* | 18513→20585 | 2073 | 690 | *** | 22066→24138 | * | * | - | - | *** | # | * | * | - | - |
| 24 | *pkip* | 20634←21143 | 510 | 169 | *** | 24187←24696 | * | * | - | - | *** | # | * | * | - | - |
| 25 | *ac25* | 21183←22133 | 951 | 316 | *** | 24736←25686 | * | * | 0.999 | 0.997 | *** | # | * | * | # | # |
| 26 | *ac26* | 22209→22598 | 390 | 129 | *** | 25762→26151 | * | * | - | - | *** | # | * | * | - | - |
| 27 | *iap-1* | 22600→23460 | 861 | 286 | *** | 26153→27013 | * | * | - | - | *** | # | * | * | - | - |
| 28 | *lef-6* | 23465→23986 | 522 | 173 | *** | 27018→27539 | * | * | - | - | *** | # | * | * | - | - |
| 29 | *ac29* | 24046←24261 | 216 | 71 | *** | 27599←27814 | * | * | - | - | *** | # | * | * | - | - |
| 30 | *ac30* | 24315←25706 | 1392 | 463 | *** | 27868←29259 | * | * | 0.998 | 0.998 | *** | # | * | * | # | # |
| 31 | *sod* | 25820→26275 | 456 | 151 | *** | 29373→29828 | * | * | - | - | *** | # | * | * | - | - |
| **hr2** |  | 26293-26961 | 669 |  |  | 29846-30643 | 798 |  | 0.795 |  |  | # | # |  | # |  |
| 32 | *fgf* | 27041←27586 | 546 | 181 | *** | 30723←31268 | * | * | - | - | *** | # | * | * | - | - |
| 33 | *Ac-Hisp* | 27733←28281 | 549 | 182 | *** | 31415←31963 | * | * | - | - | *** | # | * | * | - | - |
| 34 | *ac34* | 28294←28941 | 648 | 215 | *** | 31976←32623 | * | * | 0.981 | 0.991 | *** | # | * | * | # | # |
| 35 | *v-ubi* | 28962→29195 | 234 | 77 | *** | 32644→32877 | * | * | - | - | *** | # | * | * | - | - |
| 36 | *39k/pp31* | 29242←30069 | 828 | 275 | *** | 32924←33751 | * | * | - | - | *** | # | * | * | - | - |
| 37 | *lef-11* | 30063←30401 | 339 | 112 | *** | 33745←34083 | * | * | - | - | *** | # | * | * | - | - |
| 38 | *ac38* | 30364←31014 | 651 | 216 | *** | 34046←34696 | * | * | - | - | *** | # | * | * | - | - |
| 39 | *p43* | 31078←32169 | 1092 | 363 | *** | 34760←35851 | * | * | - | - | *** | # | * | * | - | - |
| 40 | *p47* | 32177←33382 | 1206 | 401 | *** | 35859←37064 | * | * | - | - | *** | # | * | * | - | - |
| 41 | *lef-12* | 33381→33926 | 546 | 181 | *** | 37063→37608 | * | * | - | - | *** | # | * | * | - | - |
| 42 | *gta* | 34010→35530 | 1521 | 506 | ***gta truncated*** | 37692→38858 | 1167 | 388 | 0.730 | 0.700 | *** | 37692→39212 | * | * | 0.996 | 0.993 |
| 43 | *ac43* | 35544→35777 | 234 | 77 | ***se-lef8 truncated*** | 38690←40483 | 1794 | 597 |  |  | *** | 39226→39459 | * | * | - | - |
| 44 | *ac44* | 35758→36153 | 396 | 131 |  |  |  |  |  |  | *** | 39440→39835 | * | * | - | - |
| 45 | *ac45* | 36155→36733 | 579 | 192 |  |  |  |  |  |  | *** | 39837→40415 | * | * | 0.998 | - |
| 46 | *odv-e66* | 36718→38832 | 2115 | 704 |  |  |  |  |  |  | *** | 40400→42514 | * | * | - | - |
| 47 | *trax-like* | 38938←39204 | 267 | 88 |  |  |  |  |  |  | *** | 42620←42886 | * | * | - | - |
| 48 | *ac48* | 39278←39619 | 342 | 113 |  |  |  |  |  |  | *** | 42960←43301 | * | * | - | - |
| 49 | *pcna* | 39643←40413 | 858 | 285 |  |  |  |  |  |  | *** | 43325←44182 | * | * | 0.999 | - |
| 50 | *lef-8* | 40523←43153 | 2631 | 876 |  |  |  |  |  |  | *** | 44205←46835 | * | * | 0.9999 | 0.999 |
| 51 | *dnaj domain protein* | 43180→44136 | 957 | 318 |  |  |  |  |  |  | *** | 46862→47818 | * | * | 0.999 | 0.997 |
| 52 | *ac52* | 44339←44710 | 372 | 194 |  |  |  |  |  |  | *** | 47809←48393 | * | * | - | - |
| 53 | *ac53* | 44712→45131 | 420 | 139 |  |  |  |  |  |  | *** | 48395→48814 | * | * | 0.998 | 0.993 |
| 53a | *lef-10* | 45128→45364 | 237 | 78 |  |  |  |  |  |  | *** | 48811→49047 | * | * | - | - |
| 54 | *vp1054* | 45222→46319 | 1098 | 365 | ***se111*** | 40504→41751 | 1248 | 415 |  |  | *** | 48905→50002 | * | * | 0.999 | 0.997 |
| 55 | *ac55* | 46411→46632 | 222 | 73 |  |  |  |  |  |  | *** | 50094→50315 | * | * | - | - |
| 56 | *ac56* | 46634→46888 | 255 | 84 |  |  |  |  |  |  | *** | 50317→50571 | * | * | - | - |
| 57 | *ac57* | 47073→47558 | 486 | 161 |  |  |  |  |  |  | *** | 50756→51241 | * | * | 0.992 | - |
| 58 | *ac58* | 47574←47747 | 174 | 57 |  |  |  |  |  |  | ***ac58/59*** | **51257←51775** | **519** | **172** |  |  |
| 59 | *ac59* | 47882←48091 | 210 | 69 |  |  |  |  |  |  |  |  |  |  |  |  |
| 60 | *ac60 chab-like* | 48103←48366 | 264 | 87 |  |  |  |  |  |  | *** | 51787←52050 | * | * | - | - |
| 61 | *fp* | 48513←49157 | 645 | 214 |  |  |  |  |  |  | *** | 52197←52841 | * | * | - | - |
| **hr2a** |  | 48679-48708 | 30 |  |  |  |  |  |  |  |  | 52363-52392 | * |  | - |  |
| 62 | *lef-9* | 49184→50734 | 1551 | 516 |  |  |  |  |  |  | *** | 52868→54418 | * | * | 0.999 | 0.996 |
| 63 | *ac63* | 50795→51262 | 468 | 155 |  |  |  |  |  |  | *** | 54479→54946 | * | * | - | - |
| 64 | *gp37/p34.8* | 51283←52191 | 909 | 302 | ***se-iap3 truncated***  ***/vAcRev-iap3*** | 41811←42704 | 894 | 297 |  |  | *** | 54967←55875 | * | * | - | - |
| 65 | *DNA-pol* | 52329←55283 | 2955 | 984 |  |  |  |  |  |  | *** | 56013←58967 | * | * | - | - |
| 66 | *ac66* | 55292→57718 | 2427 | 808 |  |  |  |  |  |  | *** | 58976→61402 | * | * | - | - |
| 67 | *lef-3* | 57721←58878 | 1158 | 385 |  |  |  |  |  |  | *** | 61405←62562 | * | * | 0.999 | - |
| 68 | *ac68* | 58720→59298 | 579 | 192 |  |  |  |  |  |  | *** | 62404→62982 | * | * | - | - |
| 69 | *mtase* | 59276→60064 | 789 | 262 | ***mtase truncated*** | 42824→43339 | 621 | 206 | 0.776 | 0.737 | *** | 62960→63748 | * | * | - | - |
| 70 | *hcf-1* | 60110→60982 | 873 | 290 | *** | 43385→44257 | * | * | - | - | *** | 63794→64666 | * | * | - | - |
| 71 | *iap-2* | 61016→61765 | 750 | 249 | *** | 44291→45040 | * | * | - | - | *** | 64700→65449 | * | * | - | - |
| 72 | *ac72* | 61824→62006 | 183 | 60 | *** | 45099→45281 | * | * | - | - | *** | 65508→65690 | * | * | - | - |
| 73 | *ac73* | 62015←62314 | 300 | 99 | *** | 45290←45589 | * | * | - | - | *** | 65699←65998 | * | * | - | - |
| 74 | *ac74* | 62311←63108 | 798 | 265 | *** | 45586←46383 | * | * | 0.999 | - | *** | 65995←66792 | * | * | # | - |
| 75 | *ac75* | 63126←63527 | 402 | 133 | *** | 46401←46802 | * | * | - | - | *** | 66810←67211 | * | * | - | - |
| 76 | *ac76* | 63543←63797 | 255 | 84 | *** | 46818←47072 | * | * | - | - | *** | 67227←67481 | * | * | - | - |
| 77 | *vlf-1* | 63813←64952 | 1140 | 379 | *** | 47088←48227 | * | * | - | - | *** | 67497←68636 | * | * | - | - |
| 78 | *ac78* | 64958←65287 | 330 | 109 | *** | 48233←48562 | * | * | - | - | *** | 68642←68971 | * | * | - | - |
| 79 | *ac79* | 65290←65604 | 315 | 104 | *** | 48565←48879 | * | * | 0.997 | 0.990 | *** | 68974←69288 | * | * | # | # |
| 80 | *gp41* | 65607←66836 | 1230 | 409 | *** | 48882←50111 | * | * | - | - | *** | 69291←70520 | * | * | - | - |
| 81 | *ac81* | 66826←67527 | 702 | 233 | *** | 50101←50802 | * | * | - | - | *** | 70510←71211 | * | * | - | - |
| 82 | *tlp* | 67376←67918 | 543 | 180 | *** | 50651←51193 | * | * | 0.998 | 0.994 | *** | 71060←71602 | * | * | # | # |
| 83 | *p95* | 67884→70427 | 2544 | 847 | *** | 51159→53702 | * | * | - | 0.990 | *** | 71568→74111 | * | * | - | # |
| **hr3** |  | 70468-71133 | 666 |  |  | 53743-54408 | * |  | 0.997 |  |  | 74152-74817 | * |  | # |  |
| 84 | *ac84* | 71165→71731 | 567 | 188 | *** | 54440→55006 | * | * | 0.998 | - | *** | 74849→75415 | * | * | # | - |
| 85 | *ac85* | 71934→72095 | 162 | 53 | *** | 55209→55370 | * | * | - | - | *** | 75618→75779 | * | * | - | - |
| 86 | *pnk/pnl* | 72131←74215 | 2085 | 694 | *** | 55406←57490 | * | * | - | - | *** | 75815←77899 | * | * | - | - |
| 87 | *p15* | 74356→74736 | 381 | 126 | *** | 57631→58011 | * | * | - | - | *** | 78040→78420 | * | * | - | - |
| 88 | *cg30* | 74737←75531 | 795 | 264 | *** | 58012←58806 | * | * | - | - | *** | 78421←79215 | * | * | - | - |
| 89 | *vp39* | 75534←76577 | 1044 | 347 | *** | 58809←59852 | * | * | - | - | *** | 79218←80261 | * | * | - | - |
| 90 | *lef-4* | 76596→77990 | 1395 | 464 | *** | 59871→61265 | * | * | - | - | *** | 80280→81674 | * | * | - | - |
| 91 | *ac91* | 77987←78661 | 675 | 224 | *** | 61262←61933 | 672 | 223 | 0.996 | 0.973 | *** | 81671←82342 | # | # | # | # |
| 92 | *p33* | 78699←79478 | 780 | 259 | *** | 61971←62750 | * | * | - | - | *** | 82380←53159 | * | * | - | - |
| 93 | *ac93* | 79477→79962 | 486 | 161 | *** | 62749→63234 | * | * | - | - | *** | 83158→83643 | * | * | - | - |
| 94 | *odv-e25* | 79971→80657 | 687 | 228 | *** | 63243→63929 | * | * | 0.994 | 0.996 | *** | 83652→84338 | * | * | # | # |
| 95 | *helicase* | 80694←84359 | 3666 | 1221 | *** | 63966←67631 | * | * | 0.996 | 0.998 | *** | 84375←88040 | * | * | # | # |
| 96 | *ac96* | 84346→84867 | 522 | 173 | *** | 67618→68139 | * | * | - | - | *** | 88027→88548 | * | * | - | - |
| 97 | *ac97* | 84839→85009 | 171 | 56 | *** | 68111→68281 | * | * | - | - | *** | 88520→88690 | * | * | - | - |
| 98 | *38k* | 85021←85983 | 963 | 320 | *** | 68293←69255 | * | * | - | - | *** | 88702←89664 | * | * | - | - |
| 99 | *lef-5* | 85918→86715 | 798 | 265 | *** | 69190→69987 | * | * | - | - | *** | 89599→90396 | * | * | - | - |
| 100 | *p6.9* | 86712←86879 | 168 | 55 | *** | 69984←70151 | * | * | - | - | *** | 90393←90560 | * | * | - | - |
| 101 | *p40v* | 86921←88006 | 1086 | 361 | *** | 70193←71278 | * | * | - | - | *** | 90602←91687 | * | * | - | - |
| 102 | *p12* | 88026←88394 | 369 | 122 | *** | 71298←71666 | * | * | - | - | *** | 91707←92075 | * | * | - | - |
| 103 | *p48* | 88375←89538 | 1164 | 387 | *** | 71647←72810 | * | * | - | - | *** | 92056←93219 | * | * | - | - |
| 104 | *vp80* | 89564→91639 | 2076 | 691 | *** | 72836→74911 | * | * | - | - | *** | 93245→95320 | * | * | - | - |
| 105 | *he65* | 91667←93328 | 1662 | 553 | *** | 74939←76600 | * | * | - | - | *** | 95348←97009 | * | * | - | - |
| **hr4a** |  | 93456→93605 | 150 |  |  | 76728→76877 | * |  | - |  |  | 97137→97136 | * |  | - |  |
| 106 | *ac106* | 93873→94058 | 186 | 62 | ***ac106/107*** | **76947→77678** | **732** | **243** |  |  | **#** | **97356→98087** | **#** | **#** |  |  |
| 107 | *ac107* | 94059→94391 | 333 | 110 |  |  |  |  |  |  |  |  |  |  |  |  |
| 108 | *ac108* | 94392←94709 | 318 | 105 | *** | 77679←77996 | * | * | 0.997 | - | *** | 98088←98405 | * | * | # | - |
| 109 | *ac109* | 94721←95893 | 1173 | 390 | *** | 78008←79180 | * | * | 0.997 | - | *** | 98417←99589 | * | * | # | - |
| 110 | *ac110* | 95929←96099 | 171 | 56 | *** | 79216←79386 | * | * | 0.988 | 0.982 | *** | 99625←99795 | * | * | # | # |
| 111 | *ac111* | 96148←96351 | 204 | 67 | *** | 79435←79638 | * | * | - | - | *** | 99844←100047 | * | * | - | - |
| 112 | *ac112* | 96521→96784 | 264 | 87 | ***ac112/113*** | **79808→80584** | **777** | **258** |  |  | **#** | **100217→100993** | **#** | **#** |  |  |
| 113 | *ac113* | 96789→97298 | 510 | 169 |  |  |  |  |  |  |  |  |  |  |  |  |
| **hr4b** |  | 97396-97881 | 486 |  |  | 80682-81093 | 412 |  | 0.823 |  |  | 101091-101502 | # |  | # |  |
| 114 | *ac114* | 97886←99160 | 1275 | 424 | *** | 81098←82372 | * | * | 0.998 | 0.998 | *** | 101507←102781 | * | * | # | # |
| 115 | *pif-3* | 99182←99796 | 615 | 204 | *** | 82394←83008 | * | * | 0.998 | - | *** | 102803←103417 | * | * | # | - |
| 116 | *ac116* | 99804←99974 | 171 | 56 | *** | 83016←83186 | * | * | - | - | *** | 103425←103595 | * | * | - | - |
| 117 | *ac117* | 99910→100197 | 288 | 95 | *** | 83122→83409 | * | * | - | - | *** | 103531→103818 | * | * | - | - |
| 118 | *ac118* | 100231←100704 | 474 | 157 | *** | 83443←83916 | * | * | - | - | *** | 103852←104325 | * | * | - | - |
| 119 | *pif-1* | 100699→102291 | 1593 | 530 | *** | 83911→85503 | * | * | 0.998 | 0.996 | *** | 104320→105912 | * | * | # | # |
| 120 | *ac120* | 102296→102544 | 249 | 82 | *** | 85508→85756 | * | * | 0.992 | 0.988 | *** | 105917→106165 | * | * | # | # |
| **hr4c** |  | 102606-102635 | 30 |  |  | 85818-85847 | * |  | - |  |  | 106227-106256 | * |  | - |  |
| 121 | *ac121* | 102647→102823 | 177 | 58 | *** | 85859→86035 | * | * | - | - | *** | 106268→106444 | * | * | - | - |
| 122 | *ac122* | 102713←102901 | 189 | 62 | *** | 85925←86113 | * | * | - | - | *** | 106334←106522 | * | * | - | - |
| 123 | *pk2* | 102964←103611 | 648 | 215 | *** | 86176←86823 | * | * | 0.998 | 0.995 | *** | 106585←107232 | * | * | # | # |
| 124 | *ac124* | 103793→104536 | 744 | 247 | *** | 87005→87748 | * | * | - | - | *** | 107414→108157 | * | * | - | - |
| 125 | *lef-7* | 104553←105233 | 681 | 226 | *** | 87765←88445 | * | * | - | - | *** | 108174←108854 | * | * | - | - |
| 126 | *chitinase* | 105282←106937 | 1656 | 551 | *** | 88494←90149 | * | * | 0.986 | 0.989 | *** | 108903←110558 | * | * | # | # |
| 127 | *v-cath* | 106983→107954 | 972 | 323 | *** | 90195→91166 | * | * | - | - | *** | 110604→111575 | * | * | - | - |
| 128 | *gp64* | 108179←109717 | 1593 | 530 | *** | 91391←92983 | * | * | 0.999 | - | *** | 111800←113392 | * | * | # | - |
| 129 | *p24* | 109900→110496 | 597 | 198 | *** | 93112→93708 | * | * | - | - | *** | 113521→114117 | * | * | - | - |
| 130 | *gp16* | 110524→110844 | 321 | 106 | *** | 93736→94056 | * | * | - | - | *** | 114145→114465 | * | * | - | - |
| 131 | *PE/pp34* | 110903→111661 | 759 | 252 | *** | 94115→95083 | 969 | 322 | 0.768 | 0.771 | *** | 114524→115492 | # | # | # | # |
| 132 | *ac132* | 111873→112532 | 660 | 219 | *** | 95086→95745 | * | * | - | - | *** | 115495→116154 | * | * | - | - |
| 133 | *alk-exo* | 112560→113819 | 1260 | 419 | *** | 95773→97032 | * | * | 0.999 | 0.998 | *** | 116182→117441 | * | * | # | # |
| 134 | *94k* | 113870←116281 | 2412 | 803 | *** | 97083←99494 | * | * | 0.999 | 0.999 | *** | 117492←119903 | * | * | # | # |
| 135 | *p35* | 116492→117391 | 900 | 299 | ***tn10 truncated*** | 99804→100181 | 378 | 125 |  |  | # | 120213→120590 | # | # |  |  |
|  |  |  |  |  | ***iso-is10r*** | 100419→101012 | 594 | 197 |  |  | # | 120828→121421 | # | # |  |  |
|  |  |  |  |  | ***cat*** | 101260→101919 | 660 | 219 |  |  | # | 121669→122328 | # | # |  |  |
| hr5 |  | 117479..117987 | 506 |  |  | 102170..102678 | 509 |  |  |  |  | 122579..123087 | # |  |  |  |
| 136 | *p26* | 118044→118766 | 723 | 240 | *** | 102735→103457 | * | * | - | - | *** | 123144→123866 | * | * | - | - |
| 137 | *p10* | 118839→119123 | 285 | 94 | *** | 103530→103814 | * | * | 0.996 | 0.989 | *** | 123939→124223 | * | * | # | # |
| 138 | *p74-pif* | 119135←121072 | 1938 | 645 | *** | 103826←105763 | * | * | 0.997 | 0.995 | *** | 124235←126172 | * | * | # | # |
| 139 | *me53* | 121205←122554 | 1350 | 449 | *** | 105896←107245 | * | * | 0.997 | 0.993 | *** | 126305←127654 | * | * | # | # |
| 140 | *ac140* | 122625→122807 | 183 | 60 | *** | 107316→107498 | * | * | - | - | *** | 127725→127907 | * | * | - | - |
| 141a | *ie-01* | Join  (122832→122945,  127150→128946) | 1911 | 636 | *** | Join  (107523→107636,  111838→113634) | 1911 | 636 | 0.999 | 0.998 | *** | Join  (127932→128045,  132247→134043) | # | # | # | # |
| 141 | *ie0* | 122832→123617 | 786 | 261 | *** | 107523→108308 | * | * | - | - | *** | 127932→128717 | * | * | - | - |
| 142 | *49k* | 123632→125065 | 1434 | 477 | *** | 108323→109756 | * | * | 0.999 | 0.998 | *** | 128732→130165 | * | * | # | # |
| 143 | *odv-e18* | 125069→125341 | 273 | 90 | *** | 109758→110030 | * | * | - | - | *** | 130167→130439 | * | * | - | - |
| 144 | *odv-ec27* | 125357→126229 | 873 | 290 | *** | 110046→110918 | * | * | - | - | *** | 130455→131327 | * | * | - | - |
| 145 | *ac145* | 126299→126532 | 234 | 77 | *** | 110987→111220 | * | * | - | - | *** | 131396→131629 | * | * | - | - |
| 146 | *ac146* | 126527←127132 | 606 | 201 | *** | 111215←111820 | * | * | - | - | *** | 131624←132229 | * | * | - | - |
| 147 | *ie-1* | 127198→128946 | 1749 | 582 | *** | 111886→113634 | * | * | - | - | *** | 132295→134043 | * | * | - | - |
| 148 | *odv-e56* | 129008←130138 | 1131 | 376 | *** | 113696←114826 | * | * | - | - | *** | 134105←135235 | * | * | - | - |
| 149 | *ac149* | 130167←130490 | 324 | 107 | *** | 114855←115178 | * | * | - | - | *** | 135264←135587 | * | * | - | - |
| 150 | *ac150* | 130456→130755 | 299 | 99 | *** | 115144→115443 | * | * | - | - | *** | 135553→135851 | * | * | - | - |
| 151 | *ie-2* | 130857←132083 | 1227 | 408 | *** | 115545←116771 | * | * | - | - | *** | 135954←137180 | * | * | - | - |
| 152 | *ac152* | 132109←132387 | 279 | 92 | *** | 116797←117075 | * | * | - | - | *** | 137206←137484 | * | * | - | - |
| 153 | *pe38* | 132526→133491 | 966 | 321 | *** | 117214→118179 | * | * | 0.999 | 0.997 | *** | 137623→138588 | * | * | # | # |
| 154 | *ac154* | 133591→133836 | 246 | 81 | *** | 118279→118524 | * | * | - | - | *** | 138688→138933 | * | * | - | - |

^1^ ORF number of AcMNPV; ^2^ Name of the ORF in other NPV; ^3^ ORF’s position and direction in the corresponding virus; ^4^ homology ORF and amino acid sequence identity to AcMNPV; - 100% of identity; * represent the content was in accordance with the corresponding location content of AcMNPV; # represent the content was in accordance with the corresponding location content of vAcRev-1; The underscore character represent these genes were unique to vAcRev-1; Extrabold represent these genes were typical to vAcRev-1 and vAcRev-2.
